# Exploring Ion Channel Magnetic Pharmacology: Are Magnetic Cues a Viable Alternative to Ion Channel Drugs?

**DOI:** 10.1002/bies.202400200

**Published:** 2024-12-09

**Authors:** Vitalii Zablotskii, Tatyana Polyakova, Alexandr Dejneka

**Affiliations:** ^1^ Institute of Physics of the Czech Academy of Sciences Prague Czech Republic

**Keywords:** cell membrane, ion channel drug, ion channels, magnetic cues, magnetic field, magnetic nanoparticles

## Abstract

We explore the potential of using magnetic cues as a novel approach to modulating ion channel expression, which could provide an alternative to traditional pharmacological interventions. Ion channels are crucial targets for pharmacological therapies, and ongoing research in this field continues to introduce new methods for treating various diseases. However, the efficacy of ion channel drugs is often compromised by issues such as target selectivity, leading to side effects, toxicity, and complex drug interactions. These challenges, along with problems like drug resistance and difficulties in crossing biological barriers, highlight the need for innovative strategies. In this context, the proposed use of magnetic cues to modulate ion channel expression may offer a promising solution to address these limitations, potentially improving the safety and effectiveness of treatments, particularly for long‐term use. Key developments in this area are reviewed, the relationships between changes in ion channel expression and magnetic fields are summarized, knowledge gaps are identified, and central issues relevant to future research are discussed.

## Graphical abstract

**Ion channel magnetic pharmacology**. Mechanosensitive ion channels and calcium signaling might be regulated using moderate static uniform and gradient magnetic fields. It also appears possible to manipulate the activity of voltage‐gated channels and cytosolic Ca^2^⁺ levels with low‐frequency magnetic fields.

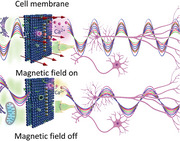

## 1 Introduction

A living cell functions as a complex electrical system that includes charges, ions, currents, electric fields, and interactions, present at all levels of cellular organization [1, 2]. Since a magnetic field (MF) acts on moving electric charges (currents), and a non‐uniform (gradient) magnetic field also affects “*non‐magnetic*” (diamagnetic) intracellular components, it is not surprising that we can observe a wide variety of biomagnetic effects both at the cellular level and at the organism level [3]. Starting from ions like potassium, sodium, calcium, and chlorine, and moving on to the basic building blocks of life (DNA, actin, microtubules, and proteins), up to the membranes of cellular organelles and the outer cell membrane, distributed electric charges and their interactions play a crucial role in the mechanisms of many if not most, biochemical processes. The continuous coordinated work of thousands of ion channels sets the membrane potential (electrical voltage), which, by definition, represents the difference in electric potentials arising between the charges on the inner and outer sides of the cell membrane. It is a fact that not only the outer cell membrane (from ‐5 mV to ‐100 mV) has a membrane potential, but also the membranes of mitochondria (‐140 mV), the endoplasmic reticulum (‐45 mV), the nucleus (‐15 mV), lysosomes (+19 mV), and phagosomes (+27 mV) [1]. At equilibrium, the magnitude of the membrane potential is determined not only by the balance of ion fluxes (mainly K^+^, Na^+^, and Cl^−^ ions) but also depends on the state of the cell, its age, and its interactions with neighboring cells through gap junction channels.

The typical number of ion channels on a cell membrane is thousands of ion channels per square micrometer, depending on the cell type, size, and functional specialization. It is important to note that the energy required to open or close ion channels barely exceeds the energy of thermal fluctuations, *k_B_T* (where *k_B_* is the Boltzmann constant, and *T* is the absolute temperature in K). Indeed, the Coulomb forces governing the opening of an ion channel are on the order of *F* = 10 pN [4]. Taking into account the radius of the ion channel *r* = (0.5–1) nm, the energy required to change the state of the ion channel can be estimated as ∆W≈*F*∙*r* = (5–10) pN·nm, which slightly exceeds the energy of thermal fluctuations, *k*_B_ *T*  =  4.1 pN·nm. Comparing these two energy values, it becomes clear that even a small amount of energy from magneto‐mechanical stress in the cell membrane can significantly influence the likelihood of ion channels opening or closing [5]. This, in turn, changes the activity of membrane ion channels and the membrane potential. In other words, smaller mechanical shear stress in a cell membrane may affect cell functionality by influencing the opening and closing of mechanosensitive channels. For example, a small shear stress from fluid flow (0.1–0.3 Pa) is able to activate individual TRPV4 channels [6]. In endothelial cells, exposure of the ion channels P2 × 4, TRP, and Piezo1 to mechanical or magnetic stresses of 1.5 Pa mediates the influx of extracellular calcium [7, 8].

It is particularly important to emphasize the high sensitivity of ion channels in endothelial cells to shear stress generated by blood flow through arteries and capillaries. In endothelial cells, very small shear stresses (less than 1 Pa or 0.00750 mmHg) can induce changes in the expression of mechanosensitive ion channels. This is 16,000 times smaller than the radial blood pressure in arteries, which is 120/80 mmHg. In the case of applying a magnetic field, an additional shear stress appears in the membranes of endothelial cells, which can act either in the same direction or in the opposite direction to the shear stress created by the flow of arterial blood. This physical mechanism is key to regulating the activity of ion channels using external magnetic fields [9]. Thus, to achieve magnetic control over the activity of ion channels, it is necessary to create magneto‐mechanical shear stress of several Pascals in cellular membranes. In general, the profound influence of combined electrical‐mechanical coupling allows for magnetic control over the operation of ion channels in cells [10].

### 1.1 Magnetic Regulation of Cell Membrane Potential

**FIGURE 1:**
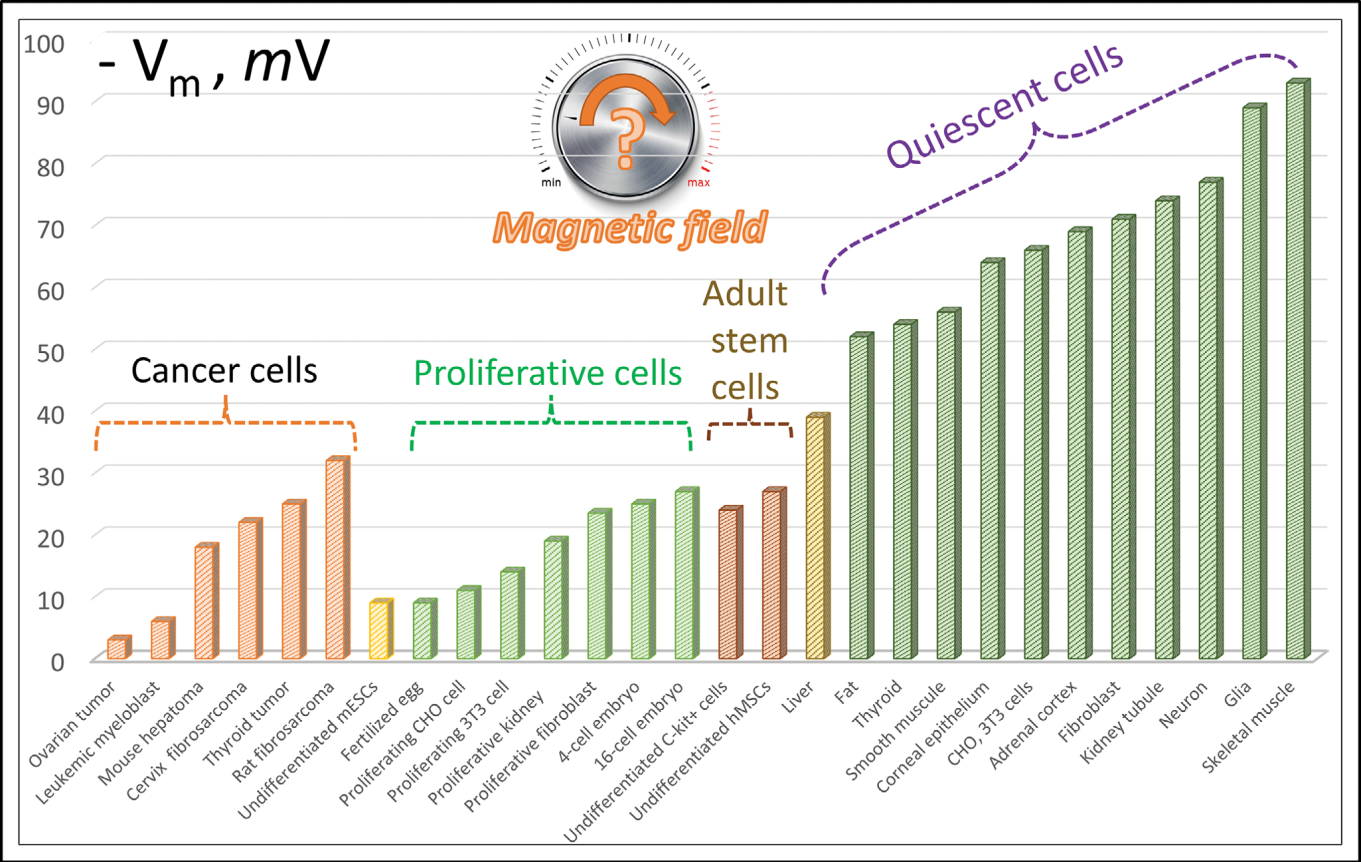
Membrane potentials (in mV) for different cell types [13]. The potential values were taken from [14, 15] and references therein. At the top, a magical tuning knob is shown that adjusts the membrane potential through a magnetic field. The question is: Can the cell membrane potential be regulated using a magnetic field?

The magnitude of the cell membrane potential can serve as a direct, quantitative indicator of ion channel activity. Figure 1 shows the distribution of cells by membrane potential. Interestingly, cells characterized by a certain average membrane potential (*V*_m_) form three large domains: cancer cells (3 mV <│*V*_m_│<30 mV), proliferative and stem cells (7 mV <│*V*_m_│<27 mV), and quiescent cells with │*V*_m_│>50 mV. The possibility of remotely controlling membrane potential opens new, exciting perspectives for cell biology and regenerative medicine [11]. The importance of the ability to regulate membrane potential is demonstrated, for example, by the fact that membrane voltage controls the exit from pluripotency and the onset of germ layer differentiation in the embryo [12].

One can imagine a magical tuning knob (Figure 1) that, when rotated, allows us to adjust the magnetic field and, consequently, alter the membrane potential, thereby controlling the process of cell differentiation [16] and modifying their phenotype [17].

A groundbreaking idea here could be to increase the membrane potential (making *V_m_* more negative) of cancer cells using a magnetic field, as these cells paradoxically have a low membrane potential (Figure 1), which determines their high invasiveness. It is implied that the electrostatic energy of the cell membrane, which determines the membrane's mechanical stiffness and its ability to change shape, is proportional to the square of the magnitude of the membrane potential [18]. We speculate that increasing the membrane potential of cancer cells using a magnetic field could significantly reduce their invasive capacity. For example, exposure of cancer cells MDA‐MB‐231, BT‐549, and MDA‐MB‐468 to a gradient rotating magnetic field (0.4 T and 5 Hz) significantly inhibits cell invasion and migration [19, 20] Intriguingly, membrane depolarization (a less negative *V_m_*) is related to the emergence and maintenance of cancer stem cells, giving rise to sustained tumor growth [21]. Thus, ion channel‐dependent magnetic regulation of cell membrane potential and cancer progression represent a new paradigm in cancer treatment. This is consistent with the recently introduced concept of ion channel pharmacology and cancer electroceuticals [22].

The underlying mechanism of magnetic regulation of the membrane potential is as follows: A non‐uniform or sufficiently strong magnetic field can cause a redistribution of ion fluxes and molecules that affect the cell membrane potential by creating a specific microscale spatial concentration pattern. Depending on the strength, gradient magnitude, and direction of the magnetic field, this can either attenuate the opening and closing of voltage‐gated ion channels or block the channels, thereby drastically altering both the resting and action membrane potentials. In the absence of a magnetic field, the resting membrane potential is accurately described by the Nernst equation, where 𝑉_𝑚_ is determined by the logarithm of the ion concentration ratio across the membrane. However, when a static magnetic field is applied, the Nernst equation is modified by adding a term that is proportional either to the magnitude of the magnetic field gradient [18] or to the modulus of magnetic field induction [23]. When cells are exposed to an alternating magnetic field, 𝑉_𝑚_ is proportional to the derivative of the magnetic induction with respect to time [24].

### 1.2 How to Drive Ion Channels With Static and Alternative Magnetic Fields

Ion channels play a key role in regulating the electrical activity of cells and many physiological processes, such as nerve impulse conduction, muscle contractions, and hormone secretion. Modulating the activity of ion channels can significantly aid in the treatment of various diseases. For example, blocking or activating specific ion channels can be an effective strategy for treating neurological disorders, cardiovascular diseases, and cancer [25, 26]​. However, only a few antibody drugs targeting ion channels are currently in early clinical trials, out of the 650 ion channel‐targeting drugs in development [25, 27]. At the same, drugs acting on ion channels lack high target selectivity, which often leads to multiple side effects.

On the other hand, a static magnetic field (0.14 T applied for 72 h) regulates T‐type calcium ion channels and mediates human mesenchymal stem cell proliferation [28]. A static moderate (a few tesla) magnetic field with a sufficiently high enough gradient (>1 kT/m) is predicted to generate mechanical stress in the cell membrane and activate both stretch‐activated and voltage‐gated ion channels through changes in the resting membrane potential [29] An uniform static magnetic field (400 mT) causes an increase in membrane potential of nerve fibers in *Metapenaeus ensis* shrimps [30].

The presence of magnetic biogenic nanoparticles (BMNs) or artificial nanoparticles on the cell membrane significantly facilitates the control of ion channels. In this case, biogenic magnetic nanoparticles found in the cells of many organs [8] and iron ion clusters in human dopaminergic neurons [31] act as mediators of mechanical stresses and pressures induced by the magnetic field. For example, the modulation of the ion channel activity (e.g., for P2 × 4, TRP, Piezo1) can be achieved when the magnetic pressure is equal to the activation/saturation pressure of different types of the mechanosensitive ion channels in the membranes of endothelial cells [8]. Since in cancer, several mechanosensitive ion channels are overexpressed [32, 33], one can speculate that an application of static gradient magnetic field in combination with BMNs is capable of down‐regulating their expression.

**FIGURE 2:**
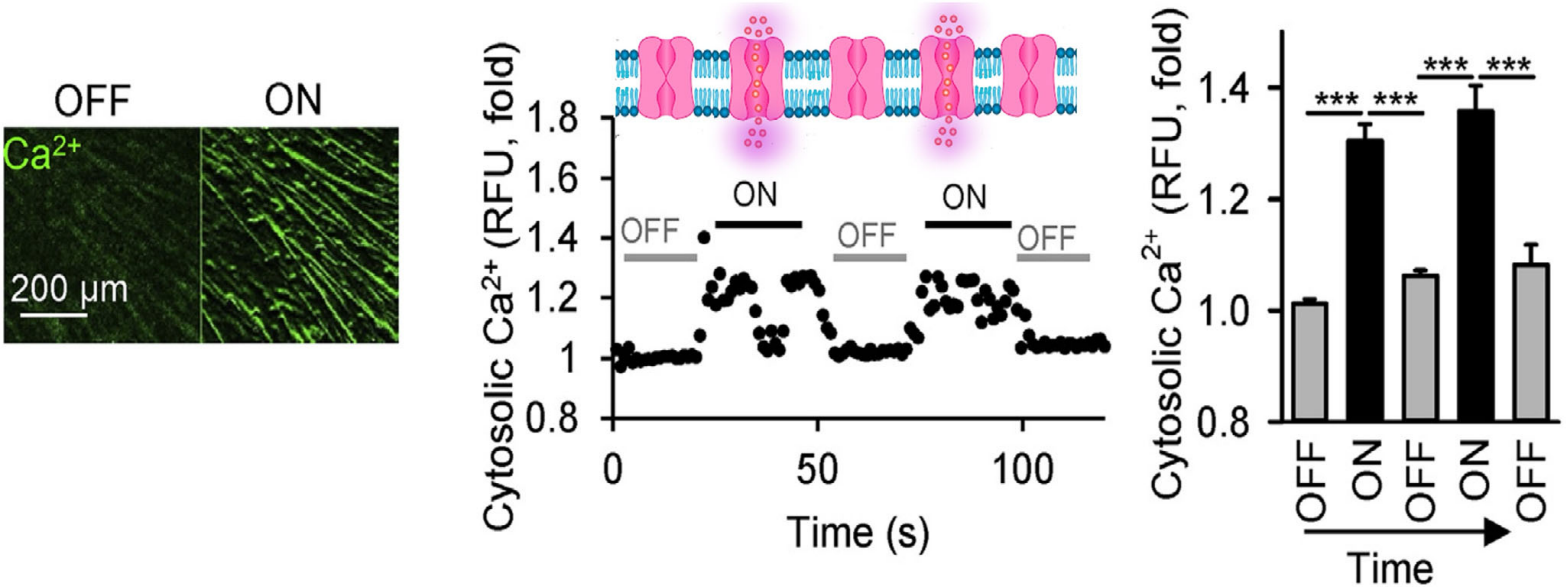
Ion channel opening and increase in cytosolic Ca^2+^ in C2C12 myotubes occur under the application of pulses of a 70 mT magnetic field with a frequency of 10 Hz. The application of magnetic field pulses also triggers actin polymerization (on the left). The cytosolic Ca^2+^ levels reverted to basal values each time the alternating magnetic field was turned off (on the right). Reproduced with permission from [24].

More pronounced effects of the magnetic field on ion channels are observed when a time‐varying magnetic field is applied. Exposure of skeletal muscle cells to a spatiotemporally modulated magnetic field (70 mT and 1–10 Hz frequency) triggers the activation of voltage‐gated sodium channels, resulting in an 8‐mV change in membrane potential, which causes a significant increase in cytosolic Ca^2+^ levels, leading to actin polymerization [24]. Here, the most pronounced result is the change in cytosolic Ca^2+^ levels under pulses of a 70 mT magnetic field with a frequency of 1–10 Hz (Figure 2). These findings open the door to utilizing alternating magnetic fields to boost the function of skeletal muscles in myopathies.

**FIGURE 3:**
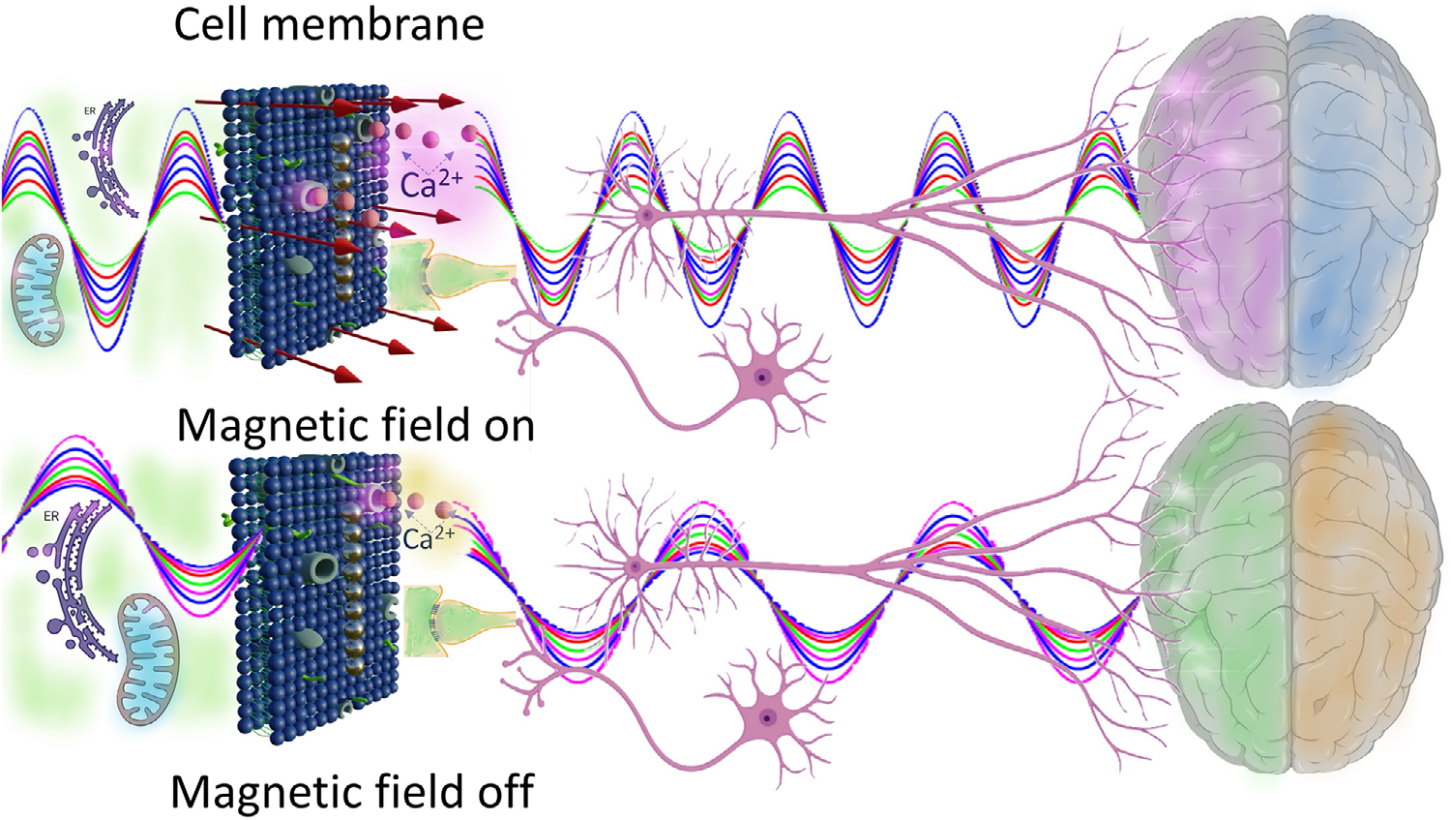
Modulation of intracellular (left) and extracellular (right) calcium waves with a magnetic field, which generates mechanical stress on the cell membrane through a chain of BMNs. A low‐frequency alternating magnetic field changes both the amplitude and frequency of calcium waves [9].

A theoretical model [9] predicts a possibility of the modulation of calcium ion channel activity and calcium signaling in the endothelium through the application of either a time‐varying or static gradient magnetic field. The most intriguing result here is an opportunity for magnetic‐field‐controlled frequency modulation of calcium waves and magnetic switching between the calcium decoders ultimately impacting protein synthesis, which could enable the control of various cellular processes, including cell proliferation, differentiation, and migration. Figure 3 illustrates schematically how intracellular extracellular calcium waves can be altered by a low‐frequency (0.5–140 mHz) magnetic field, which generates mechanical stress on the cell membrane through a chain of BMNs. Importantly, the operating frequencies of calcium signal decoders such as NF‐kB, MAPK, NFAT, and Glycogen Phosphorylase Kinase (GPK) lie within the same frequency range: 0.5 to 140 mHz [34].

Summarizing, it is important to note that despite the large number of scientific articles dedicated to the biological effects of magnetic fields, there are still few experiments that establish a quantitative relationship between ion channel activity and changes in membrane potential on one hand, and the parameters of the magnetic field and exposure time on the other. The problem is that many experimental results in the literature have not been analyzed in terms of their potential impact on ion channel activity. Most efforts have been focused on analyzing gene expression changes caused by magnetic fields. However, changes in gene expression can also occur due to alterations in the electrical activity of ion channels and the cell's membrane potential. Indeed, genetic networks are influenced by bioelectrical signals [35, 36]. These bioelectric signals are regulated by the proteins that form membrane ion channels. These signals may impact genetic pathways through the transport of calcium and various signaling molecules, electrically‐induced conformational changes in the voltage‐sensing domains of membrane proteins, and the activation of voltage‐gated channels [37, 38, 39]. The fact that endogenous electric forces can influence gene expression should not be surprising, as DNA carries a negative electric charge with a surface density of (100–200) mC/m^2^.[1, 40] Moreover, external magnetic forces acting on unwinding DNA can alter the rate of DNA synthesis by generating additional supercoils in its structure [41, 42]. Further studies on the effects of magnetic fields on ion channel activity, membrane potential, bioelectric signal propagation, and their decoding are necessary to better understand the mechanisms behind electromagnetically induced changes in gene expression.

### 1.3 Open Questions and Challenges

A key question about the efficacy of magnetic cues remains unresolved: can magnetic cues reach the same therapeutic effectiveness as ion channel‐targeting drugs? Answering this question would require a direct comparison between the efficacy of magnetic cues and that of drugs acting on the same ion channels. Such a comparison may become possible in the future through in vivo experiments conducted under controlled physiological conditions.

An important question in ion channel magnetic pharmacology is whether advanced technology and equipment are necessary to regulate ion channel expression. Technologies enabling minimally invasive magnetic control of ion channel activity are essential for successful implementation. For example, a nanomaterials‐based toolkit (m‐Torquer) allows for magnetic field‐controlled modulation of intracellular calcium ions in neurons [43]. Additionally, a magnetic system capable of generating well‐controlled homogeneous and gradient magnetic fields, as outlined in [44], is required for focusing magnetic fields and precisely guiding nanoparticles.

Unlike pharmacological agents, a magnetic field can penetrate the cell nucleus without obstruction, which presents a challenge, as it may affect mechanosensitive channels on the nuclear membrane due to the differing magnetic susceptibilities of the nucleus, cytoplasm, and cell membrane [45]. However, the possibility of magnetic control over nuclear mechanosensitive channels has not yet been discussed in the literature, making it an important topic for future research.

## 2 Conclusions

The mechanism of ion channel gating by a magnetic field is based on the fact that the probability of an ion channel being in an open state depends on membrane strain energy [46]. When magnetic nanoparticles are located on the cell membrane near an ion channel, the opening/closing mechanism should function precisely. For instance, in a rotating magnetic field, torque forces generated by a 200 nm magnetic nanoparticle can mechanically open bound Piezo1 channels, thereby modulating specific Ca^2+^ influx [43]. A gradient magnetic field applied to two iron‐loaded ferritin particles can activate mechanosensitive ion channels to which the ferritin particles are coupled [47]. Even in the absence of magnetic nanoparticles, the expression of mechanosensitive ion channels can be driven by a focused high‐gradient magnetic field [29].

Although we have gathered data on specific examples of MF‐mediated regulation of ion channel expression, there remains a significant gap in systematic experimental studies that analyze membrane ion channel activity and changes in membrane potential under similar conditions in both homogeneous and gradient magnetic fields. This gap makes it difficult to draw definitive conclusions. However, our preliminary concept of developing a new research direction—ion channel magnetic pharmacology—appears promising and warrants further exploration.

The advantages of using magnetic cues over traditional pharmacological agents are evident: magnetic fields are non‐invasive and can penetrate deeply into the human body without obstruction. Furthermore, modern advancements in magnetic systems may allow for focused targeting of specific organs or injury sites and the precise administration of magnetic nanoparticles [44]. Finally, using magnetic fields to regulate ion channel expression could potentially avoid the side effects commonly associated with ion channel drugs.

While much of the literature on ion channels focuses on the effects of low‐frequency (1–50 Hz) and high‐frequency (> 10 kHz) magnetic fields, it is worth considering that the frequencies of intracellular Ca^2^⁺ oscillations and the operating frequencies of proteins involved in signal decoding are specific to each cell type, ranging from about 1 mHz in endothelial cells to about 1000 mHz in cardiac cells and neurons [48, 49]. This suggests that the most intriguing effects of magnetic fields on ion channel expression, such as a resonance phenomenon, are likely to occur specifically within the millihertz range of oscillating (or rotating) magnetic fields.

## Conflicts of Interest

The author declares no conflicts of interest

## Data Availability

Data sharing is not applicable to this article as no datasets were generated or analyzed during the current study.
